# Use of NH_4_Cl for activation of carbon xerogel to prepare a novel efficacious adsorbent for benzene removal from contaminated air streams in a fixed-bed column

**DOI:** 10.1007/s40201-020-00533-5

**Published:** 2020-10-04

**Authors:** Ayoob Rastegar, Mitra Gholami, Ahmad Jonidi Jafari, Ahmad Hosseini-Bandegharaei, Majid Kermani, Yeganeh Kosar Hashemi

**Affiliations:** 1 Department of Environmental Health Engineering, School of Public Health, Sabzevar University of Medical Sciences, Sabzevar, Iran; 2 Department of Environmental Health Engineering, School of Public Health, Iran University of Medical Sciences, Tehran, Iran; 3 Research Center for Environmental Health Technology, Iran University of Medical Sciences, Tehran, Iran; 4 Department of Chemistry, Semnan University, Semnan, Iran; 5 Department of Natural Resources and Environment, Science and Research Branch, Islamic Azad University, Tehran, Iran

**Keywords:** Activation, Benzene, Carbon, NH_4_Cl, Modeling, Xerogel

## Abstract

**Background:**

Ammonium chloride as an explosive salt has proved to be a prominent activation agent for adsorbents and increase the specific surface area and volume of cavities. In this work, the ability of this substance was scrutinized for activation of carbon aerogel to prepare an efficient adsorbent for benzene removal from air streams.

**Methods:**

A carbon xerogel was fabricated from Novallac polymer and activated by ammonium chloride.The changes in structure and morphology were considered via Brunauer–Emmett–Teller (BET), scanning electron microscopy (SEM), Fourier transform infrared (FTIR), Barrett-Joyner-Halenda (BJH), and energy dispersive X-ray (EDX) analyses. Also, comprehensive studies were conducted to vouchsafe the properties of the new adsorbent for benzene removal, using a fixed-bed column mode.

**Results:**

The results showed both the successful synthesis and the suitability of the activation process. ACX possessed a higher specific surface area (1008 g/m^3^), compared to the parent carbon xerogel (CX; 543.7 g/m^3^) and organic xerogel (OX; 47 g/m^3^), as well as a higher adsorption capacity.

**Conclusion:**

NH_4_CL is a very beneficial for modifying the structure and morphology of carbon aerogel, and the dynamic behavior of the column with respect inlet benzene concentration can be explained by Yan-Nelson model.

## Introduction

Volatile Organic Compounds (VOCs) are among the most important air pollutants. These pollutants are released from motor vehicle exhausts, chemicals production industries, solvents in some paints, petrol evaporation, and organic solvents [1–3]. VOCs are divided into two categories: methane (CH_4_) and non-methane (NMVOCs). Out of these categories, non-methane compounds high volatility is due to their molecular structure which leads to their quick release to the atmosphere [4]. These compounds come into the atmosphere from different routes, such as automobiles, chemical treatment processes, fuel storage tanks, and industries like paint and varnish. These compounds in the atmosphere are known as precursors in different environmental reactions including photochemical smog, troposphere ozone, global warming, and stratospheric ozone depletion. Among these substances, benzene is a carcinogenic compound which is really harmful to human health, and long-period exposure to it has destructive effects on the immune system, the respiratory organs, and the lymph glands [5]. Therefore, emission control and removal of benzene from vapors before releasing to the atmosphere are very important [6, 7].

The adsorption process has been accepted as one of the important methods for removal of VOCs from polluted air. A number of adsorbents such as bagasse ash, carbon aerogels, zeolites, and activated carbons have been applied as adsorbent for the removal of volatile compounds from air [4, 5, 8, 9]. However, the adsorption ability of these adsorbents is reduced due to pore clogging, low specific surface area, lower selectivity, and the loss of adsorption efficiency after regeneration of the adsorbents [10]. Therefore, in the light of exploring more useful adsorbents, the constructing of carbon aerogel as adsorbent have been extensively investigated owing to their unique properties including high surface area, low density, high selectivity, and easy recovery [10]. In ddition, absorption capacity of these aerogels does not change even after a long period of time and conducting many adsorptions/desorption cycles [6, 11], so that previous studies have reported that the absorption capacities of typical carbon aerogels are usually higher than those of granulated active carbon, against the same pollutants [12, 13]. In this regard, carbon aerogels are usually prepared by monomers such as resorcinol, formaldehyde and furfuran, in a suitable solvent [14]. In order to obtain a carbon xerogel with high porosity, the drying stage is conducted by the aid of supercritical fluids, and then the carbonization process is performed [15]. However, the monomers used are expensive and the exploited method is also dangerous [16]. Therefore, to overcome these weaknesses, some researchers have reported that Novolac is a suitable alternative precursor [16], being a low-price commercial resin with high char-yield efficiency. Indeed, following a drying stage by atmospheric pressure drying (APD) technique, pyrolysis of Novolac-containing aerogels leads to the formation of carbon aerogels with significant properties [6]. Nevertheless, the use of APD process for drying aerogels results in decreased specific surface area and micro porosity [17]. This drawback can be resolved to a great extent by chemical activation with some chemicals.

Recently, a number of studies have reported that the use of ammonium chloride as an activating agent plays an important role in the activation of adsorbents for increasing the specific surface area and volume of cavities, due to its explosive properties. The produced NH_4_Cl-activated adsorbents have been used in the removal of several pollutants from water, such as methylene blue, phenol, aniline and amoxicillin [18–21]. However, so far, this substance has not been used to activate carbon aerogels for modifying their structure and increasing their surface area. Therefore, in this work, for the first time, the NH_4_Cl was exploited for modifying the structure and porosity of the carbon xerogel, merging the NH_4_Cl properties, as an activation agent, and carbon xerogel characteristics, as an adsorbent. Subsequently, the prepared NH_4_Cl-activated carbon xerogel was utilized for benzene removal from air streams and, after the optimization of adsorption and desorption conditions, its performance was compared with that of inactivated carbon xerogel one.

## Materials and methods

### Materials

The applied chemical compounds and their role in this study are as follows. Commercial Novolac resin with 9% wt. of hexamethylenetetramine was used as organic precursor. 2-propanol was employed as the solvent. Concentrated chloric acid was exploited as catalytic hydrolysis agent. NH_4_Cl was used as the activation agent of the surface.

### Preparation and activation carbon xerogel

In this study, firstly, organic xerogel (Novolac) was **s**ynthesized according to the method reported elsewhere [22]. Briefly, for preparing sol Novolac, 15 g of Novolac resin dissolved in 85 mL 2-propanol and ultrasonic agitation was used at a frequency of 20 kHz to disperse the solution for 30 min. Then, the sol was poured into a Falcon tube and was placed inside a high pressure autoclave containing low amount of 2-propanol and treated at 105 °C for 5 h. The resulting gel was dried using of atmospheric pressure drying (APD) method. Subsequently, organic xerogel was carbonized at 850 °C for 2 h under nitrogen gas flow rate of 10 mL/min to produce carbon-xerogel, using an electrical furnace. Afterward, in order to study the effect of ammonium chloride on the organic xerogel morphology, the organic aerogel was activated by immersion method. For this purpose, a 5% (*w*/*v*) solution of activation agent (NH_4_Cl) was prepared by dissolving the appropriate amount of the salt in a 100-mL portion of distilled water. An amount of 1 g of organic xerogel was added to this solution, the mixture was stirred using a shaker at 100 rpm for 2 h, and then dried at 105 °C for 24 h. In the next step, the organic xerogel impregnated with NH_4_Cl was placed in an electric furnace under nitrogen gas in the temperature 850 °C for 2 h, under the N_2_ atmosphere with a flow rate of 10 mL/min. The activated carbon xerogel (ACA) was washed several times with distilled water to remove residuals and impurities present on its surface. At the end, sample was dried for 4 h at 105 °C and was stored in a bottle for adsorption experiments.

### Characterization

In this study, the specific surface area, total pore volume, and mean pore diameter of the xerogel before and after of activation were determined using of absorption-desorption isotherm of nitrogen gas at 77 K, using Belsorb mini II instrument, and Barrett- Joyner-Halenda (BJH) method was used to determine the mesopores area and total pore volume. Furthermore, external area, and microspores volume of the samples were determined using Boer t-Plot analysis. The morphology of samples structure was determined using scanning electron microscopy (SEM instrument model MIRA III). Also, the percentage of different elements was measured using energy dispersive X-ray (EDX) analysis. Fourier transform infrared (FTIR) spectroscopy was used to identify functional groups on the surface of the xerogel before and after activation, by recording the spectra in the range of 400 to 4000 cm^−1^.

### Experimental adsorption in conditions static and dynamic

Firstly, to determine the equilibrium adsorption capacity of adsorbent before and after activation in static conditions, a certain amount of each activated carbon xerogel and carbon xerogel and organic xerogel were placed inside a desiccator vessel in the vicinity of benzene vapors. The mass of the samples were accurately weighed every 24 h, using a digital balance with a sensitivity of four decimal, in which detecting no weight change in two consecutive measurements indicates the absorption saturation.

**Fig. 1:**
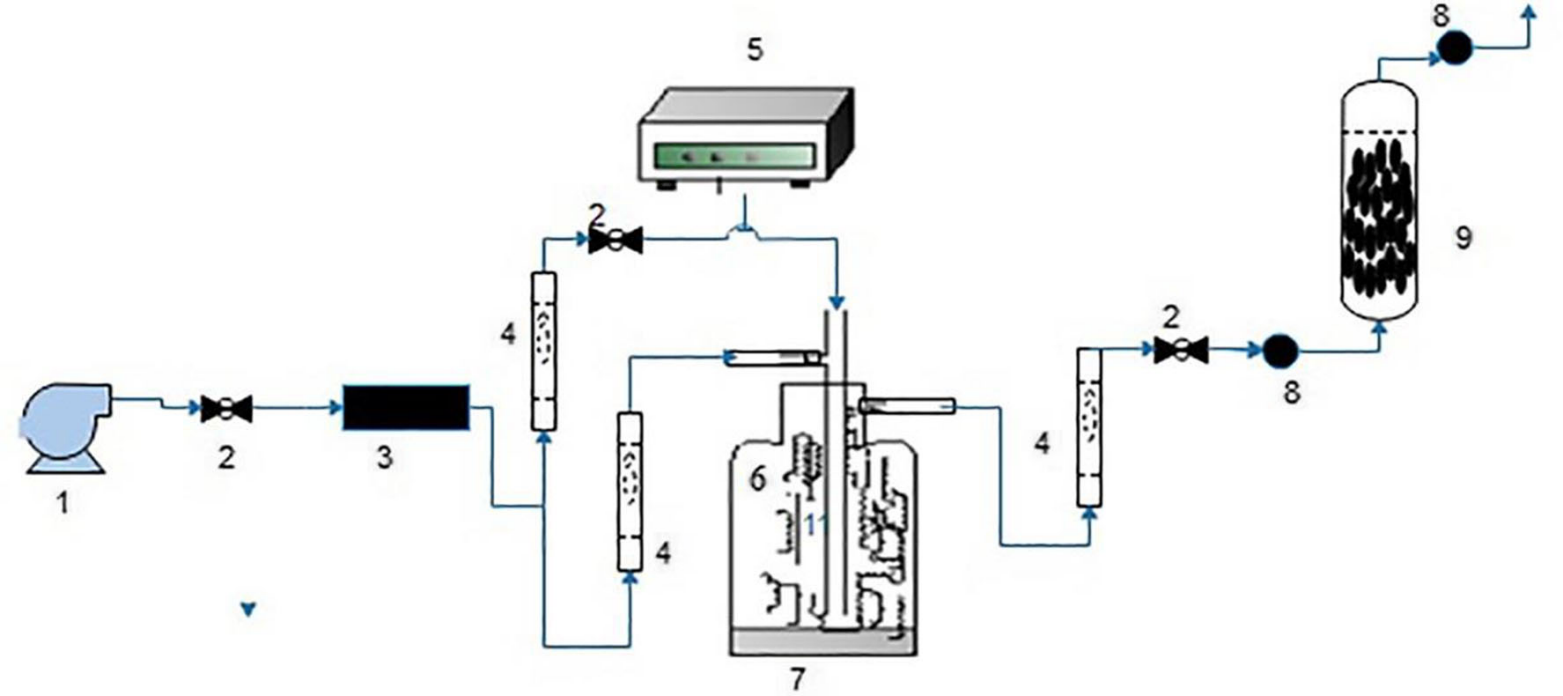
Schematic diagram of dynamic adsorption system for removal of benzene: (1) Air pump, (2)Needle valve, (3)absorbents activated carbon, (4) Rota meter, (5) Needle pump, (7) mixing chamber, (8) Sampling port, (6) Vapor benzene

The experimental adsorption in dynamic conditions was done in a fixed-bed continuous-flow reactor with an internal diameter of 1.5 cm and a height of 30 cm, having a glass gauge filter installed in the bottom of the glass column for uniform distribution of the air flow and for holding the absorbent. In this study, to provide contaminated air with different concentrations of benzene, firstly the air flow was generated by the air pump and was transferred from an activated carbon column to remove impurities. The gas stream was guided into the syringe pump path, and the amount of injected benzene liquid was controlled with using a syringe pump. Then, the benzene-containing air was introduced into a mixing chamber equipped with an electric fan to ensure uniformity. For dilution of the concentration of benzene, a certain amount of fresh air entered into the chamber from another path. To determine the efficacy of adsorbents for removal of benzene from polluted air flow, the amount of 1 g absorbents (40–60 mesh) was packed inside the column in each specified experimental stage, separately. Then, the effect of parameters such as benzene concentration (100, 200 and 400 ppmv) with flow rate 0.3 L/min were studied. The sampling from designated locations was performed by a gas tight syringe for determination of the concentrations of benzene. Then, an amount of 100 μL was injected into a gas chromatography instrument (Varian CP-3800) equipped with a FID detector. The dynamic adsorption system for removal of benzene was schematically presented in Fig. 1, at the end, the Eqs. (1 and 2) were used to assess the breakthrough- point capacity and carbon usage rate (CUR) in the breakthrough point:

1 $$ \mathrm{BC}=\frac{\left({\mathrm{C}}_{\mathrm{in}}\times {\mathrm{T}}_{\mathrm{bk}}\times \mathrm{Q}\right)}{{\mathrm{g}}_{\mathrm{adsorbent}}} $$

2 $$ \mathrm{CUR}={\mathrm{M}}_{\frac{\mathrm{adsorbent}}{\mathrm{Q}\times {\mathrm{T}}_{\mathrm{bk}}}} $$

Where BC is the amount of adsorbed benzene on a gram of absorbent to reach to breakthrough point (mg/g), C_in_ is the input concentration of benzene (mg/m), T_bk_ is breakthrough time (h), Q is air flow rate (m^3^), and M is the amount of applied absorbent (g), CUR is the absorbent usage rate in the breakthrough point.

## Results and discussion

### Characterization results

The results reported in Table 1 indicate that microstructures of OX was changed after modification processes, so that specific surface of OX after carbonization process and chemical activation with NH_4_Cl increased from 47.5 m^2^/g to 543.7 m^2^/g and 1008 m^2^/g, respectively. This increase in surface areas can be related to the formation of new micro pores, due to Novolac particles present in OX texture can decompose into volatile gases in heat treatment. In fact when these volatile gases leave the xerogel structure, new voids are generated in the xerogel composite structure [23]. Also, explosive property of NH_4_Cl in high temperature causes developing new micro pores in the texture of CX [19]. These findings can be clearly understood by considering the fact that both carbonization and activation processes can increase the surface area of the samples. Similar results were obtained by other researchers on the effect of activation and carbonization on carbon morphology [19].

**Table 1:** Characteristic properties of synthesized samples

Samples	S_BET_ (m^2^/g)	S _Meso_ (m^2^/g)	S _micro_ (m^2^/g)	S _exit_ (m^2^/g)	Pore V_Total_ (cm^3^/g)	Diameter pore (nm)	Energy constant	Monolayer volume (cm^3^ g^−1^)
OX	47,5	55.96	38.42	–	0.21	17.68	51.3	12.85
CX	543.7	625.19	74.15	38.21	0.44	3.28	239.70	143,64
ACX	1008	1247.6	1131.4	72.58	0.725	3.09	4042.5	259.93

**Fig. 2:**
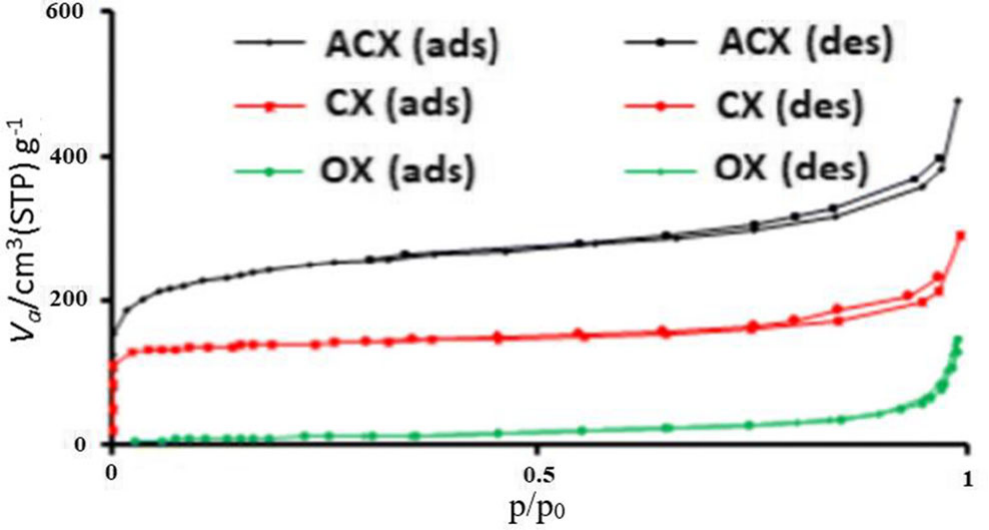
Adsorption desorption isotherms of N2 on three samples showing hysteresis

As can be seen in Fig. 2, i.e. in the adsorption - desorption isotherm of aerogel, OX displays a slow increase in the adsorption volume in low pressures, while CX and ACX samples display a fast increase in the adsorption volume in such pressures. These results can be ascribed to low micro porosity of OX, compared to CX and ACX samples. After a slight increase of N_2_ adsorption for three samples, again a sharp increase can be seen in the volume for p/p_0_ over 0.75, which may be illustrative of the existence of more mesopores [24]. Also, the higher value of energy constant in ACX case shows that the nitrogen-adsorbent interaction for this adsorbent is higher the others [25].

**Fig. 3:**
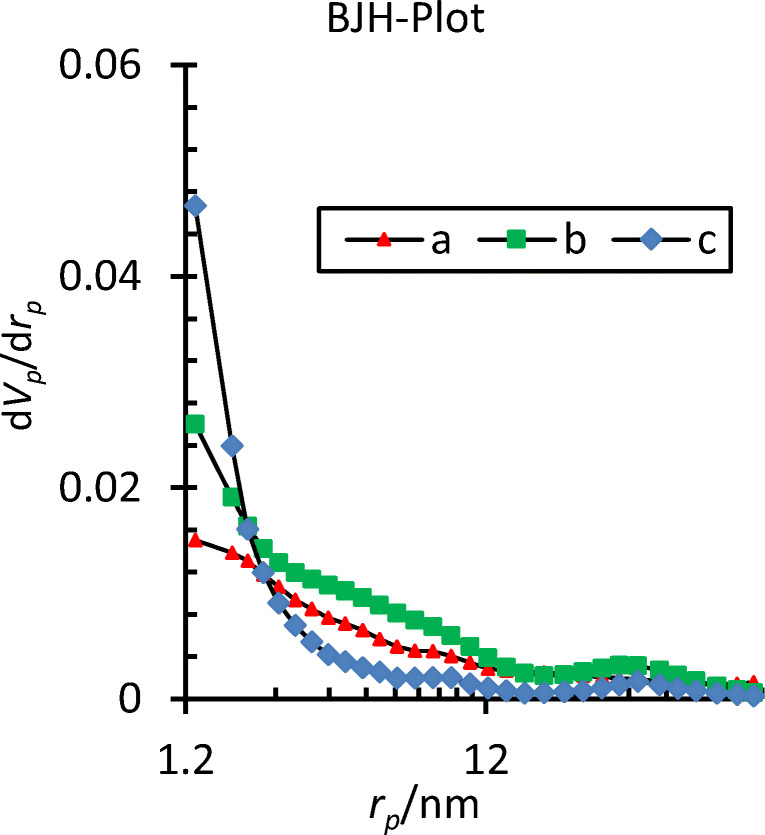
Pore size distribution of three samples based on the adsorption and desorption of N_2_ gas from the BJH plot, (a): OX (b): CX _(_c): ACX

The pore diameter distributions of three samples are showed in Fig. 3, the results showed that the pores size of samples decreased after processing with heat treatment and chemical activation. In addition, the results indicate that the adsorption/desorption isotherm is fitted with Type IV with H1-type hysteresis loop.

**Fig. 4:**
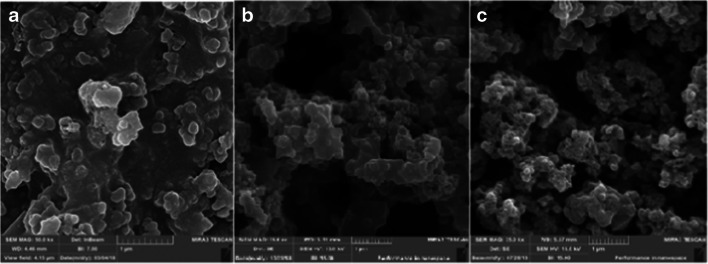
SEM image of organic xerogel (**a**), carbon xerogel (**b**), and activated carbon xerogel (**c**)

FESEM images of OX, CX_,_ and ACX samples are showed in Fig. 4. As can be seen, the surface of the samples is very inhomogeneous, and the surface of the OX is porous after both thermal and chemical activations. The size of the colloidal particles dropped after carbonization of OX and activation of CX. It can also be seen that the spherical solid clusters are distributed uniformly over the surface. Also, it can be suggested that the formation cylindrical pores in the ACX structure brings about suitable porosity for adsorption of contaminants [26].

**Fig. 5:**
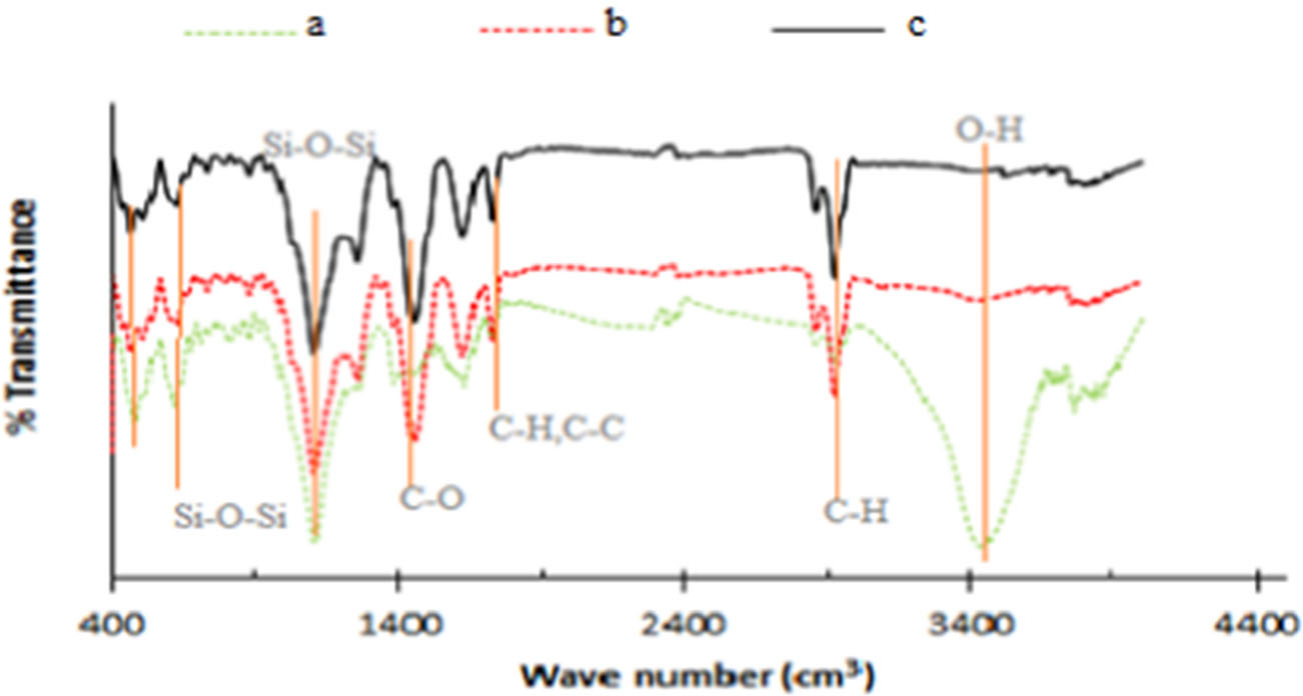
FTIR patterns of aerogel’s composite at different stages: (a) OX; (b) CX; (c) ACX

FTIR spectra of OX, CX and ACX samples were recorded to identify functional groups, which are shown in Fig. 5. The bonds related to O-H groups in the OX can be observed at 3600–3700 with high intensity, but O-H groups were not observed for CX and ACX, showing that the organic xerogel is converted from a hydrophilic substance to a hydrophobic one by thermal treatment. Moreover, the FTIR spectra show that, when the organic xerogel is modified with thermal treatment and chemical activation, significant change is created in functional groups of the OX, so that new peaks can be observed in FTIR spectra of CX and ACX, at the points of 2900 and 1450 cm^−1^ corresponding to the C-H bond, and C=C bonds, respectively. The comparison of FTIR spectra of two modified samples (i.e. CX and ACX) showed that they have similar functional groups, indicating that activating of CX with NH_4_Cl cannot cause a significant change in the chemical structure and functional groups of this sample. The FTIR results indicated that the activated carbon xerogel is rich of basic surface functional groups which in turn can help the adsorption process of a non-polar organic molecule like benzene from contaminated air steams [18].

### Adsorption of benzene in static conditions

**Fig. 6:**
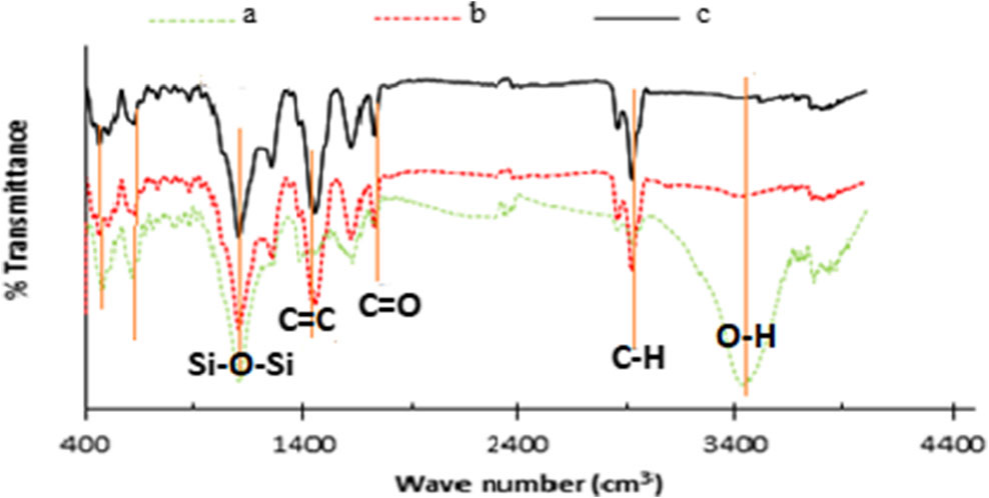
Adsorption capacities of the samples for benzene in the static conditions

The results of equilibrium adsorption capacity of benzene by OX, CX, and ACX are illustrated in Fig. 6. It was seen that the adsorption capacities has the order of OX<CX < ACX. The insignificant absorption capacity (16 mg/g) of the OX can be due to the presence of hydrophilic groups within the network and its low surface area of the organic aerogel, which cause reduction of the adsorption of benzene molecules in the adsorption sites. The increase in benzene adsorption capacity observed for by CX and ACX can be attributed to the hydrophobicity of the these adsorbent and their higher surface area [10]. The results indicate that the adsorption capacity amount of benzene was increased to more than 2 times after activation of carbon xerogel with NH_4_Cl, which is attributable to the remarkable increase in the surface area during modification. Furthermore, the amount of absorption capacity of ACX (2166 mg/g) is more than the other absorbents, such as activated carbon (375.96 mg/g), pure silica aerogel (559.26 mg/g), and porous carbon beads (1467 mg/g). In fact, it seems that there is a direct correlation between the corresponding specific surface areas and amounts of benzene absorption [8] and, from this regard, the results of this study are consistent with the results of other studies.

### Removal of benzene by CX and ACX

**Fig. 7:**
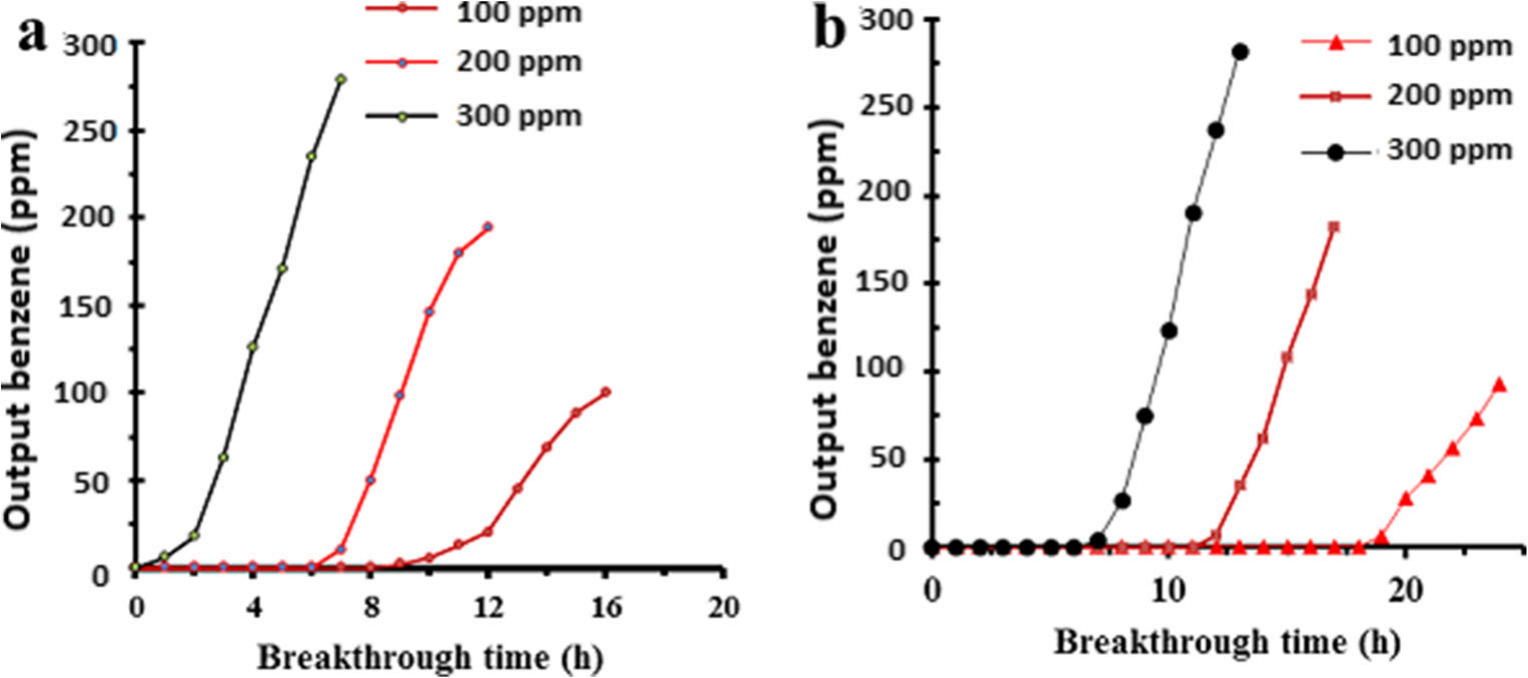
The breakthrough Curves of benzene adsorption onto (**a**) CX (**b**) ACX (Operating conditions: EBCT = 1.66 s; benzene concentration = 100, 200, 400 ppmv; absorbent dosage = 1 g and gas flow rate of 0.3 L/min

In this study, CX and ACX were more studied for considering their dynamic adsorption performance in dynamic conditions. The breakthrough time of CX and ACX for removal of benzene was studied at different inlet concentrations (100, 200, and 400 ppmv). The findings in Fig. 7a indicate that, by on creasing inlet concentration from 100 ppmv to 200 and 400 ppmv, the breakthrough time of the benzene onto CX diminished from 10 h to 7 and 1 h, respectively. In addition, it can be seen from Fig. 7b that, for the same inlet concentrations, the breakthrough time for ACX absorbent decreased from 19 h to 12 and 6 h, respectively.

The decrease of breakthrough time with the increasing the benzene concentration can be due to a further increase in the pollutant mass [27], and the decrease in the adsorption by reactive sites in the column bed [28].Comparing the studied adsorbents CX and ACX shows that ACX removes more benzene from the air flow in the same conditions. Thus, the results revealed that the activation of CX with ammonium chloride is a good idea to increase adsorption of benzene by aerogels.

To better describe the ability of ACX and CX for dynamic adsorption of benzene, these adsorbents were compared with one another in terms of breakthrough point capacity (BC) and carbon usage rate (CUR). According to results indicated in Table 2, for inlet benzene concentrations of 100, 200 and 400 ppmv, the sample ACX has an adsorption capacity of around 109, 138, and 206 mg-benzene/g adsorbent, respectively, while the CX sample can only have 57, 80, and 114 mg-benzene/g. In addition to these results, the CUR was much lower for ACX in comparison with CX at all concentrations studied. For example, when treating the polluted air flow with 400 ppm benzene, ACX obtained a benzene adsorption capacity of over 2 times greater than that of CX. Therefore, it can be concluded that the amount of CUR for ACX is less than CX, It means that 6 g of ACX can removal 1 m^3^ polluted air stream containing 400 ppm of benzene at the EBCT of 1.66 s and gas flow rate of 0.3 L/min. While 11.1 g of CX can removal 1m^3^ polluted air at the same condition. The higher performance of ACX, in comparison with CX adsorbent, in benzene removal from contaminated gas stream can be attributed to its higher surface area of the ACX which is the direct consequence of activation with ammonium chloride. Thus, it can obviously be concluded that the activation of CX with chemical activation by NH_4_Cl can increase its adsorption performance. The high surface area of ACX can be ascribed to the effect of explosion of ammonium chloride (NH_4_Cl) at 850 C°. This result is in agreement with the related literature. Therefore, it can be concluded that the NH_4_Cl has significant impact on raising surface area.

**Table 2:** The summary of the capabilities of CX and ACX columns for the removal of benzene

Adsorbent	Parameter	Concentration(ppmv)		
		100	200	400
CX	BT(h)	10	7	5
	BC(mg/g)	57.42	80	114
	CUR(g/m^3^)	5.5	7.9	11.1
ACX	BT(h)	19	12	9
	BC(mg/g)	109	138	206
	CUR(g/m^3^)	2.9	4.63	6.2

A number adsorbents have been developed and studied for the removal of benzene from gas flow, including aerogel silica/Merck activated carbon [11], carbonized polydopamine adsorbent [29], unimodal and bimodal silica-based mixed-metal oxides [30], Cupric Oxide Nanoparticles [31], and activated rice ash [32], and benzene adsorption capacities in the range of a few mg/g to about 1 g/g has been reported for them. Comparing the adsorption capacity achieved for the prepared ACX in this research with absorbents reported in the literature obviously reveals the excellent effectiveness of the developed adsorbent for the removal of benzene. This result is in agreement with the related literature [33].

### Modeling of the absorption by columns

The Nelson’s model was used to evaluate the breakthrough time of benzene in the CX and ACX columns. Equation (3) present Nelson’s model which has different parameters such as C_0_, C_t_, K_YN_ and τ_YN_. In this model, the value of k_YN_ and τ are estimated from the slope and intercepts of the linear plots of in [Ct/(C0/Ct)] versus time t, respectively [34]. Also, the C_0_ value shows the inlet concentration, mg/m^3^, and C_t_ expresses the outlet concentration at time t (mg/m^3^);

3 $$ \mathit{\ln}=\frac{C_t}{C_0-{C}_t}={k}_{YN}t-\uptau\ {k}_{YN} $$

The data of the Nelson model in different concentrations for both adsorbents are presented in Table 3. The values of the R2 tabulated in Table 3 indicate that the adsorption of benzene by both adsorbents well follows the Nelson model. In addition, the results reported in Table 3 show that by increasing the concentration of benzene, the k_YN_ value for both adsorbents has increased. The results reported in the Table 3 also indicate that the amount of k_YN_ for the ACX is lower for the adsorption of benzene, compared to CX, so it can be said that the mass transfer of benzene onto CX is less than onto ACX. Therefore, activation carbon xerogel with ammonium chloride has improved the benzene-adsorption kinetic.

**Table 3:** Predicted Yoon–Nelson model parameters to the experimental values of benzene adsorption onto CX and ACX, at different benzene concentration (EBCT = 1.66 s; absorbent dose = 1 g)

Adsorbent	Inlet concentration (ppm)	Yoon – Nelson model parameters		
		K_YN_ (h^−^)	τ (h)	R^2^
CX	100	0.96	12.5	0.990
	200	1,23	9.96	0.992
	400	1.39	5.7	0.992
ACX	100	0. 88	21,23	0.988
	200	1.05	15,6	0.981
	400	1.1	10,96	0.976

**Fig. 8:**
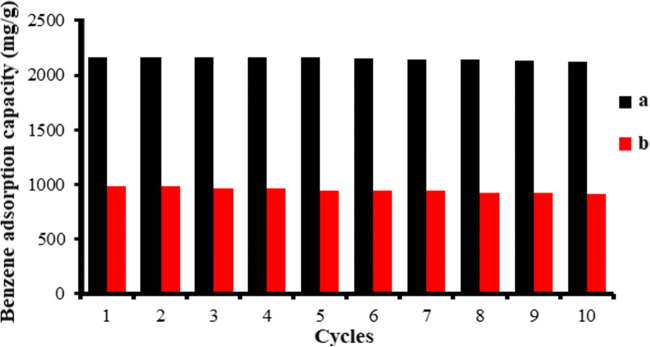
Absorption capacity of benzene by adsorbents ACX (a) and CX (b) in sequential adsorption/desorption cycles at ambient temperature and pressure

In addition, the results show that the absorbent saturation time (τ) decreased with increasing benzene concentration for CX and ACX adsorbents. These results show that, by increasing the concentration of benzene, more mass of the pollutant is absorbed into the column, and empty spaces are immediately occupied with benzene molecules. Also, from Table 3, it can be concluded that benzene absorption of contaminated air by the ACX substrate is much better than CX. Moreover, comparison of the data in Table 3 and Fig. 8 shows that there is an insignificant difference between predicted and experimental values of the breakthrough point at all inlet benzene concentrations. The results of this study is consistent with **Lin Yanyan** research on the elimination of acetaminophen using low-cost coconut shell waste pretreated with NaOH, HNO, from the aspect of the decrease observed in the correlation between the experimental and predicted K_YN_ values with increasing concentration [34].

### Regeneration studies

The absorption capacity of the ACX and CX regenerated by solvent extraction method and the results are shown in Fig. 9. The reusability potential of saturated adsorbents for removal of pollution is an important issue in the adsorption studies. As illustrated in Fig. 8, after 7 cycles, no significant changes were observed in the absorption capacity for adsorption of benzene by both adsorbents. So, it can be said that the benzene adsorbed on the ACX and CX surface can completely be desorbed and the adsorbent can successfully be regenerated. Also, this high reusability can be the consequence of the stability of porosity of the adsorbents and the possibility of complete desorption of benzene molecules from adsorbents structure. Similar studies have been reported by some researchers, like Moghadasi et al. who has reported the regeneration potential of silica aerogel and its stability for a minimum of 17 adsorption/desorption cycles, because of happening no change in the porous structure of absorbent during the process [11]. This can be because of happening no destruction in the AC/SiO_2_ cavities during the adsorption process [5].

## Conclusion

In this study, organic xerogel was synthesized using Novalak sol in dry conditions and pyrolysis at 850 °C for 2 h. Then, the process of activation of the prepared organic xerogel was carried out using NH_4_Cl as a chemical activation agent. The synthesized samples (i.e. OX, CX and ACX) had a surfaces area of 47, 543, and 1008 m^2^/g. The activated carbon xerogel had a better performance than carbon xerogel and organic xerogel in the absorption of benzene from contaminated air. For both modified adsorbents, by increasing the benzene input concentration, the value of adsorption capacity increased and the Breakthrough time decreased. The recovery of benzene from ACX and CX adsorbents was excellent and reusability studies showed that, 7 adsorption-desorption cycles crated no serious reduction in the absorption of benzene. Therefore, the obtained findings revealed that NH_4_CL was appropriate for increase of surface area of carbon-aerogels.
